# Efficacy and safety of 650 nm invasive laser acupuncture on non-specific chronic low back pain: a randomized clinical trial

**DOI:** 10.3389/fmed.2025.1579318

**Published:** 2025-05-27

**Authors:** Yejin Hong, Dongwoo Nam, Changsop Yang, Byoung-Kab Kang, Ae-Ran Kim, Kyung Min Shin, Jihye Kim, Saerom Jeon, Gwang-Cheon Park, Jae-Hong Kim

**Affiliations:** ^1^Department of Acupuncture and Moxibustion, Kyung Hee University Korean Medicine Hospital, Seoul, Republic of Korea; ^2^Department of Acupuncture and Moxibustion, College of Korean Medicine, Kyung Hee University, Seoul, Republic of Korea; ^3^KM Science Research Division, Korea Institute of Oriental Medicine, Daejeon, Republic of Korea; ^4^R&D Strategy Division, Korea Institute of Oriental Medicine, Daejeon, Republic of Korea; ^5^Digital Health Research Division, Korea Institute of Oriental Medicine, Daejeon, Republic of Korea; ^6^Korean Medicine Research, Seoul, Republic of Korea; ^7^Clinical Research Center, Dongshin University Gwangju Korean Medicine Hospital, Gwangju, Republic of Korea; ^8^Department of Acupuncture and Moxibustion Medicine, College of Korean Medicine, Dongshin University, Naju-si, Republic of Korea

**Keywords:** chronic low back pain, laser acupuncture, randomized clinical trial, integrative medicine, photobiomodulation

## Abstract

**Background:**

The study aims to determine whether 650 nm invasive laser acupuncture (ILA) is safe and effective for pain relief in patients with non-specific chronic low back pain (NSCLBP).

**Methods:**

This randomized clinical trial was conducted in two hospitals in Korea. Individuals with NSCLBP were randomized into two groups: an ILA group and a control group, in a ratio of 1:1. One hundred six participants with NSCLBP were equally assigned to the 650 nm ILA group and the control group. The 650 nm ILA group underwent 650 nm ILA for 10 min, and the control group received sham ILA for 10 min per visit, twice a week for 4 weeks, bilaterally at the GB30, BL23, BL24, and BL25 acupuncture points. All participants were trained on exercise and self-management. The primary outcome was the responder rate at the 3-day post-treatment endpoint, defined as the proportion of patients with more than a 30% reduction in NSCLBP.

**Results:**

At the 3-day post-treatment endpoint, participants in the treatment group were approximately 36% more likely to respond to the treatment than those in the control group (odds ratio 4.69; 95% confidence interval, 2.04, 10.79; *p* < 0.001), indicating a statistically significant difference in responder rates between the two groups.

**Conclusion:**

Our study provides clinical evidence for the safety and efficacy of 650 nm ILA in managing of NSCLBP. This is the first study to use ILA, which differs from previously used conventional laser acupuncture.

**Systematic review registration:**

https://cris.nih.go.kr/cris/search/detailSearch.do?search_lang=E&focus=reset_12&search_page=M&pageSize=10&page=undefined&seq=21591&status=5&seq_group=21591, identifier registration KCT0007167.

## 1 Introduction

Low back pain (LBP) is a prevalent musculoskeletal disease that affects approximately 80% of the population during their lifetimes. Within this cohort, an estimated 10%–20% develop non-specific chronic low back pain (NSCLBP), characterized by symptoms lasting longer than 12 weeks (1). NSCLBP significantly contributes to disability in daily activities, exacerbating functional limitations and resulting in disabilities. It imposes substantial financial burdens on individuals and society (2).

The World Health Organization recommends physical therapies, chiropractic manipulative therapy, and massage for chronic low back pain and advises against interventions such as lumbar belts, traction, and opioid pain killers (3). Another guidelines for NSCLBP recommend first performing conservative treatment, including low-level laser therapy (LLLT), rather than pharmacological treatment such as non-steroidal anti-inflammatory drugs (NASIDs) and muscle relaxants (4). LLLT, including laser acupuncture (LA), triggers a chemical reaction in cells called photobiomodulation or biostimulation, helping tissues heal and reducing pain (5, 6).

A recent systematic review and meta-analysis on the use of LLLT for NSCLBP, found significant benefits of pain reduction and function improvement, with few severe adverse events (SAEs) reported (7–9).

Invasive laser acupuncture (ILA) involves inserting a fiber optic-equipped acupuncture device into the body and directly irradiating the muscles and fascia internally with the laser, unlike conventional LA, which irradiates the skin’s surface using the laser (10–12). ILA is expected to produce a synergistic therapeutic effect by combining the mechanical stimulation of acupuncture with the photobiomodulation effects of laser therapy. Moreover, because the laser is emitted directly from the needle tip inserted into the tissue, it minimizes energy loss through the skin layers and enables the delivery of a sufficient amount of energy precisely to the targeted acupoints. It is a new form of laser therapy, and no prior research has been conducted on its use, apart from our pilot study.

Our pilot trial demonstrated that 650 nm ILA, with parameters including a 650 nm wavelength, 50 Hz frequency, and 20 mW power, led to notable improvements in pain and functional impairments in patients with NSCLBP and employed a study design similar to that of the current study (11).

To validate these results, a rigorous randomized clinical trial (RCT) with a larger sample size was required. We conducted this study to gather clinical data on the efficacy and safety of 650 nm ILA in treating NSCLBP.

## 2 Methods

This study received approval from the Ministry of Food and Drug Safety (Medical Device Approval No. 1322) and was registered with the Clinical Research Information Service (Registration No. KCT0007167; registration date: April 8, 2022). The first participant was enrolled on May 19, 2022, and the last participant visit and study completion occurred on August 17, 2023. This study complied with the Standard Protocol Items of the Recommendations for Interventional Trials (SPIRIT) statements (13) and followed the guidelines of the Consolidated Standards of Reporting Trials (CONSORT) (14). Detailed methods used in this study are described in a previous publication (12).

### 2.1 Study design

This study was a prospective, parallel-arm, multi-center, patient- and assessor-blinded, randomized clinical trial. A total of 106 participants were randomly assigned into either the 650 nm ILA group or the control group, with 53 participants in each group. All participants were trained on exercise and self-management. The 650 ILA group underwent 10 min of genuine 650 nm ILA treatment, while the control group received sham ILA for the same duration. The intervention was performed twice weekly for 4 weeks, targeting the following acupuncture points bilaterally: Bladder 23 (BL23; Shenshu), Bladder 24 (BL24; Qihaishu), Bladder 25 (BL25; Dachangshu) and Gallbladder 30 (GB30; Huantiao) (15, 16). These points were selected because they are commonly used in clinical practice for the treatment of low back pain and, in our pilot study, ILA applied to these points demonstrated significant pain-relieving effects in patients with chronic low back pain (11).

The primary outcome was the proportion of responders (defined as those experiencing a 30% decrease in pain, as measured by Visual Analog Scale (VAS) scores (17), without increased requirement for painkillers) at 3 days post-intervention. The secondary outcomes included changes in VAS scores, the Korean version of the Oswestry Disability Index (ODI) scores (18), and European Quality of Life Five Dimension Five Level scale (EQ-5D-5L) scores (19) at 3 days and 8 weeks post-intervention.

The study’s design is detailed in Supplementary Table 1.

### 2.2 Participant recruitment

Participants were recruited at Kyung Hee University Korean Medicine Hospital and Dongshin University Gwangju Korean Medicine Hospital in the Republic of Korea through local newspapers, posters, and hospitals/communities websites. The clinical research coordinator (CRC) provided an overview of the study to those who expressed interest during their hospital visit and obtained written informed consent before participation. During each session, the CRC explained the next visit schedule and adjusted it to ensure the participation of each participant.

### 2.3 Participation

The inclusion criteria were as follows: aged 19–70 years; NSCLBP for at least 3 months, occurring on more than 14 days per month; no use of medications for NSCLBP or stable medication (i.e., unchanged dose and type) for at least 4 weeks prior to screening; moderate pain (VAS scores ranging from 35 to 74) (20) at screening; and adequate fluency in Korean for accurate assessment completion.

The exclusion criteria were as follows: progressive radicular pain or neurological deficits; severe diseases or spinal pathologies; LBP caused by specific conditions; a record of treatment for mental illness or substance dependency within the past 6 months; moderate/severe depression (the Korean version of the Beck Depression Inventory-II score ≤ 23) (21) at screening; contraindications for ILA (e.g., blood coagulation disorders, severe skin diseases, electronic medical devices such as pacemakers); history of lumbar spinal surgery in the past year or planned during the trial; participation for social insurance or compensation purposes; simultaneous involvement in another trial; pregnancy or planning pregnancy; and being deemed unsuitable for ILA and the rescue regimen.

The dropout criteria were as follows: absence of any efficacy data after randomization (i.e., participants who did not receive the intervention or lacked baseline assessments); discontinuation by the institutional review board (IRB) or principal investigator (PI) due to inability to continue or SAE necessitating long-term treatment; withdrawal of consent; or a SAE resulting in hospitalization, surgery, serious disability, or death (Supplementary Appendix).

### 2.4 Randomization and blinding

The investigator conducted the screening interview, followed by baseline assessments by the assessor. The 106 enrolled participants were randomly assigned to either the 650 nm ILA group or the control group (53 per group). Serial numbers were generated using SAS version 9.4 (SAS Institute Inc., Cary, NC, USA) through stratified block randomization according to study sites. A combination of block sizes 4 and 6 was used depending on the number of participants at each site to ensure balanced allocation. The randomization sequence was sealed in opaque envelopes and securely stored in a dual-locked cabinet. The responsible investigator opened the envelopes and allocated participants for the intervention. The practitioner performing the intervention knew each participant’s group assignment. Due to this lack of blinding, we implemented a design where assessors and patients were blinded using sham ILA. All investigators were blinded except those administering the intervention and managing the serial numbers. Unblinding was allowed with IRB approval if necessary, such as in cases of an SAE.

### 2.5 Intervention

**FIGURE 1:**
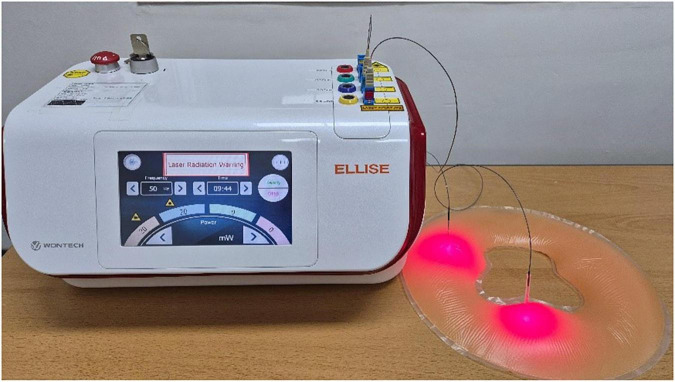
Invasive laser acupuncture device.

The treatment was administered by three board-certified Korean medical doctors specializing in acupuncture and moxibustion, each with over 10 years of clinical experience. These interventionists were affiliated with Kyung Hee University Korean Medicine Hospital and Dongshin University Gwangju Korean Medicine Hospital, and all received joint training to ensure adherence to the standardized protocol. ILA treatment used a laser-emitting device (Ellise; Wontech Co. Ltd., Daejeon, Republic of Korea) with a sterile, stainless steel, disposable acupuncture needle(external diameter, 0.3 mm; inner diameter, 0.15 mm; length, 30 mm) containing an optical fiber-coupled laser diode (InGaAIP) and a laser output device (Supplementary Figure). The ILA parameters included a frequency of 50 Hz, a power density of 63.69 W/cm^2^, an energy density of 38,216.56 J/cm^2^, an energy dose of 12 J per point, and a pulse-type wave (Figure 1).

Acupuncture needles were inserted vertically into the GB30, BL23, BL24, and BL25, with depths of 9–30 mm depending on the location (22). No manual stimulation was performed. Laser activation followed (650 ILA group: 20 mW power for 10 min; control group: 0 mW power for 10 min).

Throughout the treatment period, participants received 10-min treatments twice weekly for 4 weeks, and were trained on exercise and self-management (visit 1 to 8). Acetaminophen (500 mg) was provided for severe pain as a rescue regimen. When administered, its use was reported to the CRC and appropriately documented. At each visit, participants’ medical conditions were monitored for trial adherence. During the study period, other treatments for NSCLBP, including pharmacological treatments, physical therapy, or alternative/complementary therapies not permitted in our study, were prohibited. However, non-pharmacological and pharmacological treatments for other symptoms were allowed.

### 2.6 Outcome measurements

#### 2.6.1 Efficacy

The primary outcome was the difference in the proportion of responders between the groups, defined as participants with a greater than 30% reduction in their baseline VAS score who did not require an increase in painkiller usage at 3 days post-treatment. Secondary outcomes included changes in VAS, ODI, and EQ-5D-5L scores at 3 days and 8 weeks post-treatment.

The VAS assesses pain severity on a 0–100 scale, where higher scores indicate worse pain, and is widely used in low back pain trials. The ODI evaluates functional impairment using nine items (0–5 points each), excluding the “sexual activity” item from the original version, resulting in a total score out of 45. This raw score was converted into a percentage scale (0%–100%), with higher scores indicating greater disability (23). The EQ-5D-5L measures health-related quality of life across five dimensions, each rated on five levels. These responses were converted to a single utility score using the Korean value set, ranging from −0.066 (worst health state) to 1.000 (perfect health), with higher scores reflecting better quality of life (24).

#### 2.6.2 Safety

Adverse events (AEs) were defined as unintended and undesirable conditions occurring during or after treatment. SAEs were defined as events resulting in death, life-threatening conditions, hospitalization or its prolongation, permanent or significant disability, congenital anomalies, or other medically significant situations, as determined by the PI. The PI was responsible for SAE assessment and reporting to the IRB, which could implement trial modifications if deemed necessary. In our pilot study, there were no AEs or SAEs linked to ILA (11). Potential AEs included bleeding, hematoma, worsening pain, dizziness, and skin irritation. All AEs and SAEs were carefully recorded, noting potential causal links with the intervention, severity, timing, and corrective actions. Their incidence, frequency, and dropout rates were then compared between groups as part of the safety analysis. Vital signs were tracked at 3 days and 8 weeks post-treatment, while laboratory results were evaluated by comparing baseline values with those recorded at 3 days post-treatment and analyzing shifts between normal and abnormal ranges.

### 2.7 Sample size calculation

In our previous pilot study (11), there was a 53% difference in the responder proportions between the 650 nm ILA (93% [14/15]) and control (40% [6/15]) groups. To obtain adequate clinical data, we calculated the sample size, assuming a 30% responder proportion in the control group and 60% in the 650 nm ILA group, with a two-sided alpha level of 0.05 and a statistical power of 0.8. Based on these assumptions, a total of 84 participants (42 per group) was necessary. Accounting for a maximum dropout rate of 20%, our trial required 106 participants (53 in each group).

$$n=\frac{{\left(z_{\frac{\alpha }{2}}\,\sqrt{2\,\bar{p}\,\bar{q}}+z_{\beta }\,\sqrt{p_{t}\,q_{t}+p_{c}\,q_{c}}\right)}^{2}}{{\left(p_{t}-p_{c}\right)}^{2}}$$

$$=\frac{{\left(1.96\,\sqrt{2*0.45*0.55}+0.842\,\sqrt{0.30*0.70+0.60*0.40}\right)}^{2}}{{\left(0.60-0.30\right)}^{2}}\approx 42$$

### 2.8 Statistical analyses

The finalized data were analyzed by an independent biostatistician not involved in our trial. The primary efficacy analysis was based on the full analysis set (FAS), which included all randomized participants who received at least one treatment and provided at least one efficacy outcome including baseline data. The per-protocol set (PPS), consisting of participants who completed all scheduled treatment procedures without major protocol deviations, was used for supplementary analysis. A total of 106 participants were included in the FAS, and 99 in the PPS. The results from both sets were consistent; thus, only FAS results are presented in the main text, with PPS results available in the appendix (Supplementary Tables 2, 3). The “multiple imputation” method accounted for missing data. Statistical analyses were conducted at a significance level of 5% (two-sided) using SAS version 9.4 software (SAS Institute Inc., Cary, NC, USA). No interim analyses were conducted. Baseline characteristics and variables were compared between groups, with categorical data assessed using the chi-square or Fisher’s exact test and continuous data analyzed using either the independent *t*-test or Wilcoxon’s rank sum test. The proportion of responders were compared using Fisher’s exact test or chi-square test.

The differences in VAS, ODI, and EQ-5D-5L scores at 3 days and 8 weeks post-treatment, compared to baseline scores, between the groups were assessed using an analysis of covariance, with baseline scores serving as covariates. Within-group score changes at each time point were analyzed using a one-way analysis of variance and either a Wilcoxon signed rank test or a paired *t*-test.

During the trial, a comprehensive safety assessment monitored all SAEs and AEs. The occurrences of SAEs and AEs were compared between groups using either the Fisher’s exact test or chi-square test. Furthermore, a comparative analysis of participants with clinical laboratory test results outside the normal range was conducted between the groups.

## 3 Results

### 3.1 Participants

**FIGURE 2:**
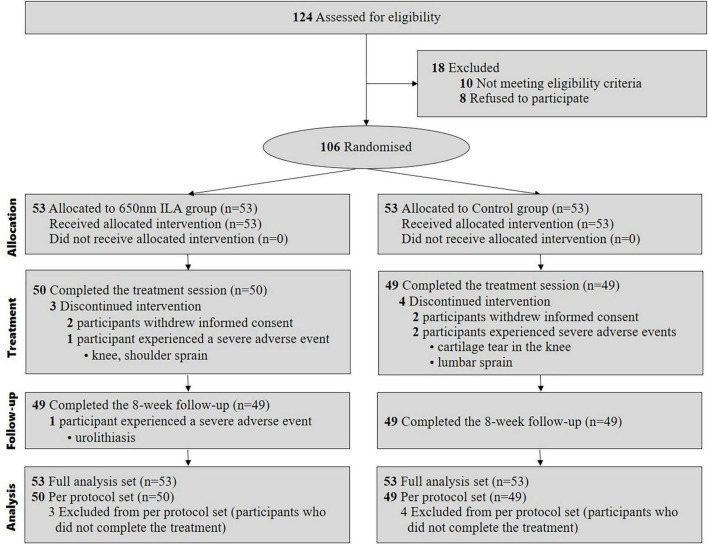
Study flow diagram.

We enrolled participants for our study between May 18, 2022, and August 17, 2023. During this period, we assessed 124 patients for eligibility, of whom 18 were excluded. Eventually, 106 patients fulfilled the inclusion criteria and were randomly assigned to either the control group (*n* = 53) or the 650 nm ILA group (*n* = 53). In both groups, four participants failed to complete the treatment; specifically, two experienced SAEs unrelated to ILA and were withdrawn from the study according to the clinical trial protocol, while the other two withdrew consent to participate due to a simple change of mind. The final analysis included data from all 106 patients (Figure 2).

### 3.2 Baseline characteristics

Table 1 shows the baseline demographic and clinical characteristics of the participants in both groups. There were no significant differences between the treatment and control groups in age, sex, medication use, or baseline scores of the VAS, ODI, and EQ-5D-5L.

**TABLE 1:** Characteristics of the participants.

Characteristics	Control (*n* = 53)	ILA (*n* = 53)	*p*-value
Age (y), mean (SD)	49.17 (15.55)	49.23 (15.54)	0.9851^a^
Sex (male/female), mean (SD)	20 (37.74)/33 (62.26)	19 (35.85)/34 (64.15)	0.8404^b^
Height (cm), mean (SD)	163.49 (8.64)	164.96 (9.49)	0.4068^a^
Weight (kg), mean (SD)	65.43 (11.25)	66.86 (11.76)	0.5227^a^
Smoking (yes/no), *n* (%)	9 (16.98)/44 (83.02)	6 (11.32)/47 (88.68)	0.4032^b^
Drinking (yes/no), *n* (%)	17 (32.08)/36 (67.92)	19 (35.85)/34 (64.15)	0.6817^b^
Medications for non-specific chronic low back pain (yes/no), *n* (%)	0/53	0/53	ND
Medications for other diseases (yes/no), *n* (%)	16 (30.19)/37 (69.81)	17 (32.08)/36 (67.92)	0.8339^b^
VAS, mean (SD)	53.92 (11.23)	56.42 (10.52)	0.2413^a^
ODI (%), mean (SD)	21.13 (10.13)	20.13 (8.8)	0.5865^a^
EQ-5D-5L, mean (SD)	0.80 (0.10)	0.78 (0.09)	0.3381^a^

^*a*^*p*-value for an independent *t*-test.

^*b*^*p*-value for a chi-square test.

### 3.2 Efficacy evaluation

#### 3.2.1 Primary outcome

At the 3-day (±1 day) post-treatment endpoint, compared to baseline, the treatment group had an approximately 36% higher likelihood (odds ratio 4.69; 95% confidence interval, 2.04, 10.79; *p* < 0.001) of response than the control group, with a statistically significant difference noted in responder rates between the two groups (Table 2).

**TABLE 2:** Comparison of the proportion of responders.

Outcome measure	ILA (*n* = 53)	Control (*n* = 53)	Responder rate difference, (95% CI)	*p*-value
Responder, *n* (%)	40 (75.47)	21 (39.62)	0.36 (0.18, 0.53)	0.0002*
Odds ratio (95% CI)	–	–	4.69 (2.04, 10.79)	

*p < 0.001, *P*-value for a chi-square test.

#### 3.2.2 Secondary outcome

There was a statistically significant difference in VAS score changes (*p* = 0.0021, visit 9; *p* = 0.0015, visit 10) between the groups, while no significant differences were observed in ODI score (*p* = 0.0839, visit 9; *p* = 0.4764, visit 10) and EQ-5D-5L score (*p* = 0.4294, visit 9; *p* = 0.7389, visit 10) changes between the groups. However, both groups showed therapeutic effects in VAS, ODI, and EQ-5D-5L changes (*p* < 0.0001) at both visit 9 (3 days ± 1 day post-treatment) and visit 10 (8 weeks ± 3 days post-treatment) (Table 3).

**TABLE 3:** Change in VAS, ODI, and EQ-5D scores at 3 days (±1 day) after the end of treatment (visit 9) and 8 weeks (±3 days) after the end of treatment (visit 10) compared to baseline.

Outcome measure	Time point	ILA (*n* = 53), mean (95% CI)	Control (*n* = 53), mean (95% CI)	Mean difference, mean (95% CI)	*p*-value
VAS	Baseline	53.92 (50.86, 57.02)	56.42 (53.52, 59.31)		
	Visit 9	29.77 (25.37, 34.17)	41.04 (36.39, 45.69)	9.84 (3.59, 16.10)	0.0021*
	Baseline - Visit 9	24.15 (20.04, 28.27)	15.37 (10.70, 20.05)		
	*p*-value^b^	<0.0001**	<0.0001**		
	Visit 10	27.87 (22.89, 32.85)	42.28 (36.79, 47.77)		
	Baseline - Visit 10	26.06 (21.13, 30.98)	14.14 (8.50, 19.77)	12.27(4.70, 19.84)	0.0015**
	*p*-value^b^	<0.0001**	<0.0001**		
ODI (%)	Baseline	21.13 (18.34, 23.93)	20.13 (17.70, 22.50)		
	Visit 9	11.46 (9.02, 13.90)	13.80 (11.89, 15.71)		
	Baseline - Visit 9	9.67 (6.63, 12.72)	6.33 (4.26, 8.40)	2.57 (−0.34, 5.48)	0.0839
	*p*-value^b^	<0.0001**	<0.0001**		
	Visit 10	11.76 (9.13, 14.40)	12.73 (10.50, 14.96)		
	Baseline - Visit 10	9.37 (6.71, 12.02)	7.39 (4.62, 10.17)	1.20 (−2.11, 4.52)	0.4764
	*p*-value^b^	<0.0001**	<0.0001**		
EQ-5D	Baseline	0.80 (0.78, 0.83)	0.78 (0.76, 0.81)		
	Visit 9	0.87 (0.85, 0.90)	0.85 (0.83, 0.87)	0.01 (−0.02, 0.04)	0.4294
	Baseline - Visit 9	0.07 (0.04, 0.10)	0.07 (0.04, 0.09)		
	*p*-value^b^	<0.0001**	<0.0001**		
	Visit 10	0.88 (0.85, 0.91)	0.86 (0.84, 0.89)	0.01 (−0.03, 0.04)	0.7389
	Baseline - Visit 10	0.08 (0.05, 0.11)	0.08 (0.05, 0.11)		
	*p*-value^b^	<0.0001**	<0.0001**		

*p < 0.01, **p < 0.001. *p*-value^a^ for ANCOVA adjusted baseline; *p*-value^b^ for comparison within group using a paired *t*-test. LS Mean, Least Squares Adjusted Mean; CI, confidence interval. †Least squares mean difference and *p*-values were analyzed using analysis of covariance (ANCOVA), with baseline scores and exercise as covariates and group as the fixed factor.

#### 3.2.3 Safety evaluation

Among the 106 clinical trial participants included in the safety evaluation, 12 (11.32%) experienced 16 adverse events. Four patients withdrew from the study due to SAEs (Supplementary Table 4). The adverse reactions included knee cartilage tear, lumbar sprain, knee sprain, shoulder sprain, and urolithiasis, with no causal relationship determined between these and the clinical study (Supplementary Table 5). Vital signs and laboratory tests showed no significant abnormal changes, remaining within normal ranges.

## 4 Discussion

This is the first clinical trial on ILA. Our study found that patients with NSCLBP in the ILA group experienced pain relief after 4 weeks, compared to the control group, suggesting ILA as an auxiliary treatment. Additionally, the clinical effectiveness and safety results provide evidence on pain management using ILA to policymakers, clinicians, and patients. Furthermore, by demonstrating the efficacy and safety of ILA, our study proposes its potential role as part of an integrated rehabilitation strategy for non-specific chronic low back pain, offering clinicians an additional modality to enhance conventional treatment approaches. This could broaden the therapeutic options available for managing chronic low back pain and contribute to more comprehensive and individualized rehabilitation strategies.

The primary outcome measured was the proportion of patients with a 30% or greater pain reduction, with an odds ratio of 4.69 indicating a significant difference. The secondary outcome (changes in VAS scores) also indicated significant improvements. After 4 weeks, the ILA group had a mean VAS score reduction of 24.15, compared to 15.37 in the control group (*p* < 0.005). The ILA group exceeded the threshold minimal clinically important difference of 20 (25) for NSCLBP on the VAS, confirming its clinical usefulness. Four weeks post-intervention, the ILA group maintained a VAS score reduction of 26.06, while the control group had a reduction of 14.14, indicating sustained effects and confirming the clinical efficacy of ILA treatment.

While the physiological mechanisms underlying low-level laser therapy (LLLT) are not fully elucidated, current evidence suggests that it may inhibit peripheral nerve conduction, promote the release of endogenous opioids (26), facilitate mast cell degranulation (27), activate serotonergic pathways (28), and exert anti-inflammatory effects by reducing edema and oxidative stress (29). Furthermore, the 650 nm wavelength specifically has been shown to modulate neural transmission by regulating acetylcholinesterase, substance P, leu-enkephalin, and c-Fos/GFAP expression (30), supporting its potential efficacy in pain relief.

The efficacy of LA for musculoskeletal pain significantly depends on energy dose. However, ILA’s efficacy may be limited by noncollimated light scattering and reflection at superficial skin layers, which impedes energy penetration. This characteristic of conventional LA may result in inadequate stimulation of acupoints, which are believed to reside within the myofascial layer of the body (31).

In response to this challenge, our study adopted a 650 nm wavelength ILA approach. Unlike conventional LA, ILA emits laser directly from acupuncture needle tip beneath the skin. This technique effectively reduces the scattering, reflection, and absorption of light as it passes through the skin, thereby improving energy delivery to the targeted acupoints. Consequently, ILA represents a promising advancement over traditional LA methods in treating musculoskeletal pain.

We designed our study to ensure that the control group received acupuncture at the same points and depth as the treatment group to achieve perfect patient blinding. The only difference was the administration of laser irradiation in the treatment group, making the control group closely resemble conventional acupuncture. Interestingly, previous research suggests that employing the same acupuncture points for controls could lead to genuine therapeutic effects (32). Our study found significant changes in VAS, ODI, and EQ-5D scores in the control group when comparing the scores before and after treatment, indicating that our clinical outcomes may be underestimated.

The VAS, which measures subjective pain intensity, tends to respond quickly to interventions, whereas functional outcomes like ODI and EQ-5D require more substantial or longer-term improvements to reach statistical significance. As participants had moderate chronic low back pain with relatively mild baseline functional disability and quality of life, the extent of measurable changes may have been limited, contributing to the differences in the statistical significance between pain and functional outcomes.

A previous study showed the ILA group improved ODI scores compared to the control group. Similarly, we observed clear functional improvements in the treatment group before and after intervention, suggesting ILA effectively enhances function.

The absence of ILA-related adverse events throughout the clinical trial led to the conclusion that ILA is a safe treatment. A study on the safety of LA indicated that it is a safe treatment; however, it mentions potential risks to organs, such as the conjunctiva and retina, from laser exposure (33). In the case of ILA, the laser is projected internally, significantly reducing such risks.

This study has limitations It focused solely on the effects of 650 nm laser acupuncture on NSCLBP, suggested to have analgesic effects in our pilot study. Additionally, since the laser-emitting device was operated by a practitioner, blinding of the operator was not feasible. Furthermore, there is a need for additional research, specifically double-blinded RCTs, to investigate the optimal parameters, including different wavelengths, energy doses, and acupoints, for ILA.

## 5 Conclusion

Our findings provide strong clinical evidence of the safety and efficacy of 650 nm ILA for managing NSCLBP, establishing a solid foundation for further research. This contributes to increasing the availability of laser therapy and promotes the development of optimal laser treatment methods for managing NSCLBP. This study could serve as a cornerstone for the use of ILA.

## Data Availability

The raw data supporting the conclusions of this article will be made available by the authors, without undue reservation.
