# Supplementary material for: Efficacy and safety of 650 nm invasive laser acupuncture on non-specific chronic low back pain: a randomized clinical trial
Source: Front Med (Lausanne). 2025 May 27;12:1579318. doi: 10.3389/fmed.2025.1579318 (PMC12149192; doi:10.3389/fmed.2025.1579318)
Supplement: Supplementary file 1 [file Supplementary_file_1.docx]

**[Supplement]**

**eTable 1. Standard Protocol Items: Recommendations for Interventional Trials (SPIRIT) Statement**

|  | Study period | | | | | | | |  |
| --- | --- | --- | --- | --- | --- | --- | --- | --- | --- |
|  | Enrollment | Allocation | Post-allocation | | | | | Close-out |  |
| Timepoint | Screening | | Visits 1–2 | Visits 3–4 | Visits 5–6 | Visits 7–8 | Visit 9 | Visit 10 |  |
| Time | Week | | 1 | 2 | 3 | 4 | 4 + 3 days | 12 |  |
| **Enrollment** | | | | | | | | | |
| Informed consent | ● |  |  |  |  |  |  |  |  |
| Sociodemographic profile | ● |  |  |  |  |  |  |  |  |
| Vital signs | ● | ● | ● | ● | ● | ● | ● | ● |  |
| Medical history | ● |  |  |  |  |  |  |  |  |
| Inclusion/exclusion criteria | ● |  |  |  |  |  |  |  |  |
| Allocation |  | ● |  |  |  |  |  |  |  |
| Visual Analogue Scale of  pain | ● |  |  |  |  |  |  |  |  |
| Beck Depression Inventory-Ⅱ test | ● |  |  |  |  |  |  |  |  |
| **Interventions** | | | | | | | | | |
| Invasive laser acupuncture (sham or 650 nm) |  |  | ● | ● | ● | ● |  |  |  |
| Education on self management and exercise |  |  | ● | ● | ● | ● |  |  |  |
| **Assessments** | | | | | | | | | |
| Changes in medical history |  |  | ● | ● | ● | ● | ● | ● |  |
| Safety assessment (incidence of AEs) |  |  | ● | ● | ● | ● | ● | ● |  |
| Clinical laboratory tests | ● |  |  |  |  |  | ● |  |  |
| Visual Analogue Scale of  pain |  |  | ● |  |  |  | ● | ● |  |
| Scores for the Korean version of the Oswestry disability index |  |  | ● |  |  |  | ● | ● |  |
| European Quality of Life  Five Dimension Five Level Scale |  |  | ● |  |  |  | ● | ● |  |

**eAppendix. Inclusion criteria, exclusion criteria, and dropout criteria**

**Inclusion Criteria**

1) Patients aged between 19 and 70 years

2) Patients with non-specific chronic low back pain (NSCLBP) that has persisted for more than 3 months and who have experienced pain for at least 14 days per month in the last 3 months before screening

a. Non-specific low back pain refers to back pain that is not associated with any specific spinal conditions or radiculopathy based on lumbar X-ray examination, medical history, physical examination, neurological examination (muscle strength, sensation, and deep tendon reflexes), and physical tests (straight leg raise test).

b. Patients who have not changed their medication regimen in the 4 weeks prior to screening; if the patient is undergoing pharmacological treatment for low back pain, the type, method, and dosage of the medication must have remained stable and unchanged for the 4 weeks prior to screening.

3) Patients who have moderate low back pain with a 100-mm Visual Analogue Scale (VAS) score between 35 and 74 at the time of screening

4) Patients who have no difficulty reading and understanding Korean

5) Patients who voluntarily sign the written informed consent form for participation in the study

**Exclusion criteria**

1) Patients with low back pain associated with radiculopathy, including radiating leg pain or neurological deficits in the lower extremities

2) Patients with serious conditions affecting the lumbar spine (malignant tumors, recent compression fractures, spinal infections, or cauda equina syndrome)

3) Patients with severe underlying diseases (cancer, severe kidney disease, severe liver disease, severe cardiovascular disease, severe neurological disease, or diabetic neuropathy)

4) Patients who have been treated for alcohol addiction, drug addiction, or serious psychiatric conditions (dementia, schizophrenia, epilepsy, or depression) within the past 6 months

5) Patients with underlying conditions that can cause back pain (trauma, ankylosing spondylitis, fibromyalgia, rheumatoid arthritis, or gout)

6) Patients for whom invasive laser acupuncture treatment should be performed with caution (blood coagulation disorders, severe skin diseases at the treatment site, the presence of metallic devices in the lumbar region, or the use of electronic medical devices such as pacemakers)

7) Patients with moderate or severe depression, as indicated by a Korean version of the Beck Depression Inventory-II score of 23 or higher

8) Patients who have undergone lumbar spine surgery in the past year or have a surgery scheduled

9) Patients who are currently pregnant or planning to become pregnant

10) Patients participating in another clinical trial

11) Patients being treated under social insurance, compensation, or indemnity coverage

12) Patients deemed unsuitable for the application of the investigational medical device or the administration of the investigational drug by the principal investigator or clinical trial coordinator

**Drop-out criteria**

1) If the patient or the patient's legal representative withdraws consent to participate in the clinical trial

2) If a serious adverse event occurs in the patient

3) If the researcher determines that it is necessary to discontinue the study due to an adverse event

4) If the patient requests to discontinue the study due to an adverse event

5) If no data is collected because the patient is never evaluated after randomization

6) If the patient cannot be followed up

7) If the researcher determines that it is not appropriate to continue the study for any other reason

| 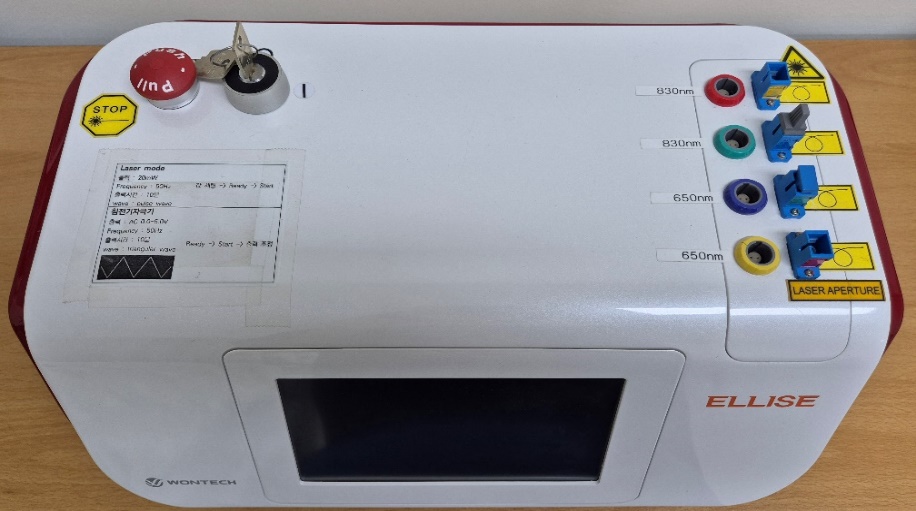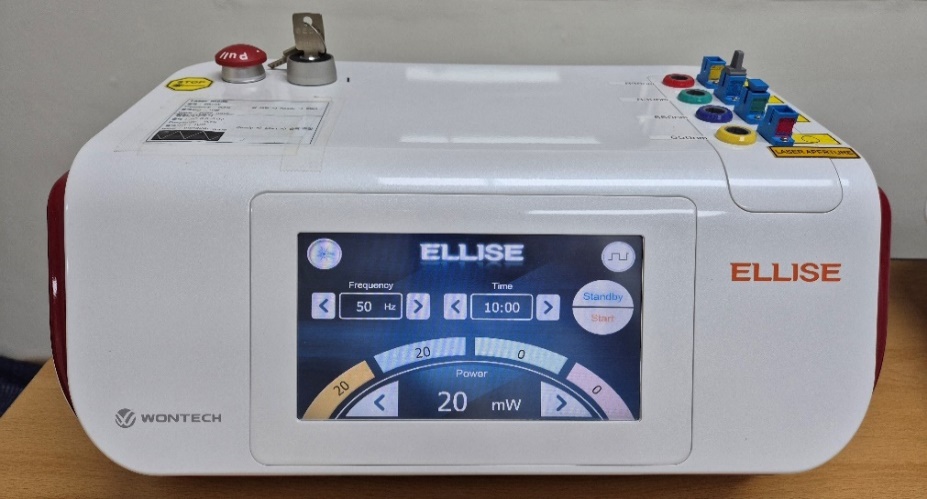  The main body |
| --- |

| 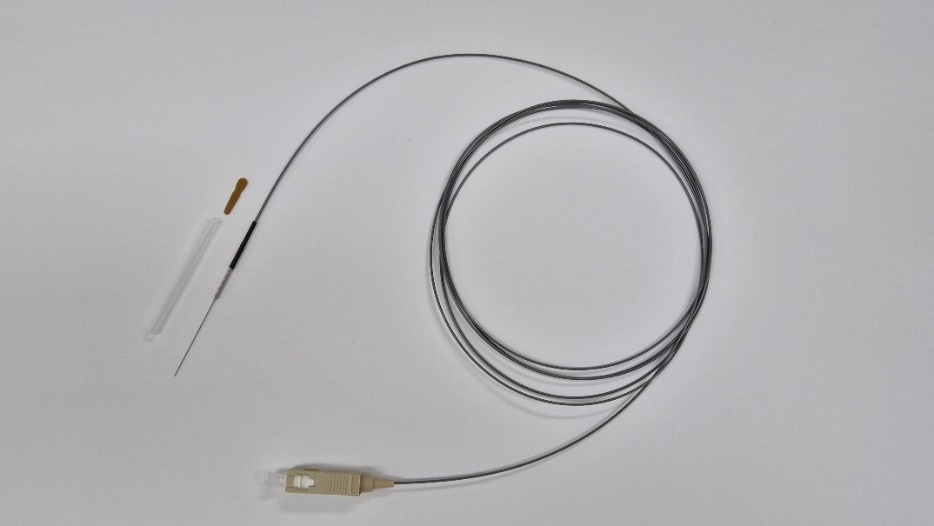  An acupuncture needle with an optical fiber-coupled laser diode  **eFigure 1.** The appearance and components of the Ellise |  |
| --- | --- |

**eTable 2. Comparison of the proportion of responders (Per protocol analysis)**

|  | **ILA (n=50)** | **Control (n=49)** | **Responder rate difference, (95% CI)** | | **p-value** |  |
| --- | --- | --- | --- | --- | --- | --- |
| Responder, n (%) | 36 (72.00) | 20 (40.82) | 0.31 (0.13, 0.50) | **0.0017*** | | |
| Odds ratio (95% CI) | - | - | 3.73 (1.61, 8.64) |  | | |

^*^p < 0.01, P-value for a chi-square test

**eTable 3. Change in VAS, ODI, and EQ-5D scores at 3 days (± 1 day) after the end of treatment (Visit 9) and 8 weeks (± 3 days) after the end of treatment (Visit 10) compared to baseline (Per protocol analysis)**

|  |  | **ILA (n = 50), mean (95% CI)** | **Control (n = 49), mean (95% CI)** | **Mean difference, mean (95% CI)** | **p-value** |
| --- | --- | --- | --- | --- | --- |
| **VAS** | **Baseline** | 54.18 (50.91, 57.45) | 56.88 (53.85, 59.90) |  |  |
|  | **Visit 9** | 29.81 (25.32, 34.30) | 41.04 (36.10, 45.98) |  |  |
|  | **Baseline - Visit 9** | 24.93 (20.45, 29.41) | 15.27 (10.84, 19.71) | 9.66 (3.23, 16.09) | **0.0036*** |
|  | **p-value^b^** | **<.0001**** | **<.0001**** |  |  |
|  | **Visit 10** | 28.11 (22.93, 33.29) | 42.25 (36.36, 48.14) |  |  |
|  | **Baseline - Visit 10** | 26.28 (20.76, 31.81) | 14.53 (9.18, 19.87) | 11.75 (3.90, 19.61) | **0.0038**** |
|  | **p-value^b^** | **<.0001**** | **<.0001**** |  |  |
| **ODI** | **Baseline** | 21.29 (18.37, 24.20) | 20.23 (17.66, 22.79) |  |  |
|  | **Visit 9** | 11.16 (8.60, 13.71) | 13.74 (11.74, 15.74) |  |  |
|  | **Baseline - Visit 9** | 9.76 (7.65, 11.87) | 6.86 (4.77, 8.94) | 2.91 (-0.11, 5.92) | 0.0586 |
|  | **p-value^b^** | **<.0001**** | **<.0001**** |  |  |
|  | **Visit 10** | 11.48 (8.69, 14.27) | 12.55 (10.25, 14.84) |  |  |
|  | **Baseline - Visit 10** | 9.47 (7.15, 11.79) | 8.22 (5.90, 10.54) | 1.25 (-2.09, 4.59) | 0.4585 |
|  | **p-value^b^** | **<.0001**** | **<.0001**** |  |  |
| **EQ-5D** | **Baseline** | 0.80 (0.77, 0.83) | 0.78 (0.76, 0.81) |  |  |
|  | **Visit 9** | 0.87 (0.85, 0.90) | 0.85 (0.83, 0.87) |  |  |
|  | **Baseline - Visit 9** | 0.08 (0.06, 0.10) | 0.06 (0.04, 0.09) | 0.01 (-0.02, 0.05) | 0.3756 |
|  | **p-value^b^** | **<.0001**** | **<.0001**** |  |  |
|  | **Visit 10** | 0.88 (0.85, 0.91) | 0.87 (0.84, 0.89) |  |  |
|  | **Baseline - Visit 10** | 0.09 (0.06, 0.12) | 0.08 (0.05, 0.11) | 0.01 (-0.03, 0.05) | 0.5990 |
|  | **p-value^b^** | **<.0001**** | **<.0001**** |  |  |

^*^ p < 0.01, ^**^ p < 0.001.

p-value^a^ for ANCOVA adjusted baseline; p-value^b^ for comparison within group using a paired t-test

LS Mean = Least Squares Adjusted Mean, CI = Confidence Interval

† Least squares mean difference and p-values were analyzed using analysis of covariance (ANCOVA), with baseline scores and exercise as covariates and group as the fixed factor.

eTable 4. Adverse Event Analysis

|  | **Control group (n = 53)** | **ILA group (n = 53)** | **p-value** |
| --- | --- | --- | --- |
| **Participants with adverse events, no. (%)** | 9 (16.98) | 3 (5.66) | 0.0659^a^ |
| **Participants with severe adverse events, no. (%)** | 2 (3.77) | 2 (3.77) | 1.0000^b^ |
| **Drop out due to a severe adverse event, no (%)** | 2 (3.77) | 2 (3.77) | 1.0000^b^ |

a: p-value for a chi-square test

b: p-value for a Fisher’s exact test

**eTable 5. Severe Adverse Event**

|  |  |  | **Severe adverse event** | **Occurence point** | **Disappearance point** | **Severity^a^** | **Causal relationship^b^** | **Treatment for adverse event^c^** | **Adverse event outcomes^d^** | **Predictability^e^** |
| --- | --- | --- | --- | --- | --- | --- | --- | --- | --- | --- |
| **Control group** |  | LA-2101 DS-R-014 | Cartilage tear in the knee | 2022-07-10 | 2022-07-25 | 4 | 5 | 4 | 1 | 2 |
|  |  | LA-2101 DS-R-080 | Lumbar sprain | 2023-08-09 |  | 4 | 4 | 4 | 3 | 1 |
| **ILA group** |  | LA-2101 DS-R-067 | Knee sprain | 2023-03-10 | 2023-03-13 | 4 | 5 | 4 | 2 | 2 |
|  |  |  | Shoulder sprain | 2023-03-10 | 2023-03-13 | 4 | 5 | 4 | 2 | 2 |
|  |  | LA-2101 DS-R-008 | Urolithiasis | 2022-07-07 | 2022-07-21 | 4 | 5 | 4 | 1 | 2 |

a: 1 mild; 2 moderate; 3 severe; 4 serious adverse events

b: 1 clearly related; 2 related; 3 possibly related; 4 not related; 5 clearly not related; 6 unknown

c: 1 none; 2 medication; 3 non-medication treatment; 4 hospitalization or prolongation of hospitalization

d: 1 recovered (no sequelae); 2 recovered (with sequelae); 3: recovering; 4 not recovered; 5 death; 6 indeterminable

e: 1 expected adverse events; 2 unexpected adverse events
